# Supplementary material for: Mating and starvation modulate feeding and host-seeking responses in female bed bugs, Cimex lectularius
Source: Sci Rep. 2021 Jan 21;11:1915. doi: 10.1038/s41598-021-81271-y (PMC7820594; doi:10.1038/s41598-021-81271-y)
Supplement: Supplementary file 1 — Supplementary Legend. [file 41598_2021_81271_MOESM1_ESM.docx]

**Mating and starvation modulate feeding and host-seeking responses in female bed bugs, *Cimex lectularius***

**Ahmed M. Saveer^1,2,*^, Zachary C. DeVries^1,3^, Richard Santangelo^1^, and Coby Schal^1,2,*^**

^1^Department of Entomology and Plant Pathology, North Carolina State University, Raleigh, North Carolina, USA;

^2^W.M. Keck Center for Behavioral Biology, North Carolina State University, Raleigh, North Carolina, USA;

^3^Present address: Department of Entomology, University of Kentucky, Lexington, Kentucky, USA

*Authors for correspondence:

Ahmed M. Saveer – Email: saveer.ahmed@gmail.com

Coby Schal – Email: coby@ncsu.edu

ORCID: Ahmed M. Saveer: 0000-0002-4587-5578; Zachary DeVries: 0000-0003-0050-7269;

Coby Schal: 0000-0001-7195-6358

**Supplementary Video S1. Expression of a female refusal behavior in *Cimex lectularius*.** Representative video recording of a 30 days starved Mated-Long female (a female was housed with a fertile male until she died) expressing ‘refusal’ behavior to protect the ectospermalege from a harassing male. The female was taken out of the experimental glass vial for a clearer recording.
